# Free breathing contrast-enhanced time-resolved magnetic resonance angiography in pediatric and adult congenital heart disease

**DOI:** 10.1186/s12968-015-0138-9

**Published:** 2015-05-22

**Authors:** Jennifer A Steeden, Bejal Pandya, Oliver Tann, Vivek Muthurangu

**Affiliations:** UCL Centre for Cardiovascular Imaging, University College London, 30 Guildford Street, London, WC1N 1EH UK; The Heart Hospital, University College London Hospital Foundation Trust, London, W1G 8PH UK; Cardiorespiratory Unit, Great Ormond Street Hospital for Children, London, WC1N 3JH UK

**Keywords:** Free-breathing, Time-resolved MR angiography, 3D stack-of-spirals, Congenital heart disease

## Abstract

**Background:**

Contrast enhanced magnetic resonance angiography (MRA) is generally performed during a long breath-hold (BH), limiting its utility in infants and small children. This study proposes a free-breathing (FB) time resolved MRA (TRA) technique for use in pediatric and adult congenital heart disease (CHD).

**Methods:**

A TRA sequence was developed by combining spiral trajectories with sensitivity encoding (SENSE, x4 *kx-ky* and x2 *kz*) and partial Fourier (75% in *kz*). As no temporal data sharing is used, an independent 3D data set was acquired every ~1.3s, with acceptable spatial resolution (~2.3x2.3x2.3mm). The technique was tested during FB over 50 consecutive volumes. Conventional BH-MRA and FB-TRA data was acquired in 45 adults and children with CHD. We calculated quantitative image quality for both sequences. Diagnostic accuracy was assessed in all patients from both sequences. Additionally, vessel measurements were made at the sinotubular junction (*N* = 43), proximal descending aorta (*N* = 43), descending aorta at the level of the diaphragm (*N* = 43), main pulmonary artery (*N* = 35), left pulmonary artery (*N* = 35) and the right pulmonary artery (*N* = 35). Intra and inter observer variability was assessed in a subset of 10 patients.

**Results:**

BH-MRA had significantly higher homogeneity in non-contrast enhancing tissue (coefficient of variance, *P* <0.0001), signal-to-noise ratio (*P* <0.0001), contrast-to-noise ratio (*P* <0.0001) and relative contrast (*P* = 0.02) compared to the FB-TRA images. However, homogeneity in the vessels was similar in both techniques (*P* = 0.52) and edge sharpness was significantly (*P* <0.0001) higher in FB-TRA compared to BH-MRA. BH-MRA provided overall diagnostic accuracy of 82%, and FB-TRA of 87%, with no statistical difference between the two sequences (*P* = 0.77). Vessel diameter measurements showed excellent agreement between the two techniques (r = 0.98, *P* <0.05), with no bias (0.0mm, *P* = 0.71), and clinically acceptable limits of agreement (-2.7 to +2.8mm). Inter and intra observer reproducibility showed good agreement of vessel diameters (r>0.988, *P*<0.0001), with negligible biases (between -0.2 and +0.1mm) and small limits of agreement (between -2.4 and +2.5mm).

**Conclusions:**

We have described a FB-TRA technique that is shown to enable accurate diagnosis and vessel measures compared to conventional BH-MRA. This simplifies the MRA technique and will enable angiography to be performed in children and adults whom find breath-holding difficult.

**Electronic supplementary material:**

The online version of this article (doi:10.1186/s12968-015-0138-9) contains supplementary material, which is available to authorized users.

## Background

Assessment of thoracic (or cardiac) anatomy is important in patients with congenital heart disease (CHD) and is one of the main indications for cardiovascular magnetic resonance (CMR) in this population [1]. Contrast enhanced MR angiography (CE-MRA) has a proven ability to detect vascular stenoses, dilation and other abnormalities [25] and is often used for this purpose. However, acquisition of high resolution, three-dimensional (3D) data is time consuming, normally taking between 10–25 s. Thus, to prevent image degradation as a result of respiratory motion, CE-MRA is generally performed during a breath-hold. Unfortunately, this limits the use of CE-MRA in small children (who are unable to comply with breath-hold instructions) and severely dyspnoeic adults. In these groups, a better approach might be to acquire each volume so quickly that respiratory motion has limited effect on image quality. This would enable CE-MRA to be performed during free-breathing and would open up this technique to a wider group of patients.

Such an approach has been partially realized by time resolved MR angiography (TRA), in which a series of volume angiograms are acquired in quick succession [7]. This technique is mainly used to provide information about perfusion kinetics [8,9], as well as to simplify scan timing in relation to the passage of the contrast bolus [10]. However, the majority of time-resolved MRA sequences use some form of data sharing across time (*e.g.* contrast-enhanced timing-robust angiography; CENTRA keyhole [11], sliding window [12], or time-resolved echo-shared angiographic technique; TREAT [13]), making them sensitive to respiratory motion artifacts. Thus, conventional TRA sequences are often performed during a breath-hold to ensure sufficient image quality.

In this study, we propose an alternative method of accelerating time resolved angiography; namely by combining time efficient spiral trajectories with sensitivity encoding (SENSE). The benefit of this approach is that there is no temporal data sharing. This may allow sufficient image quality to be achieved during free-breathing conditions. The specific aims of this study were; a) To demonstrate the feasibility of acquiring free-breathing time resolved MRA (FB-TRA) in pediatric and adult congenital heart disease, b) To quantitatively assess image quality of FB-TRA in comparison with a conventional breath-hold angiographic sequence (BH-MRA), c) To compare the diagnostic accuracy of FB-TRA to conventional BH-MRA, and d) To assess the accuracy and reproducibility of vessel measurements made from FB-TRA compared to BH-MRA.

## Methods

### Study population

Between June and July 2014, 45 consecutive children with heart disease (congenital and cardiomyopathy) and adults with congenital heart disease (32 male, 13 female) were enrolled into this study. Inclusion criteria were: a) Clinical referral for cardiac MR imaging and b) Clinically necessary CE-MRA. The exclusion criteria were general contraindications to MR, such as pregnancy or MR-incompatible implants. One further child was recruited in whom contrast administration was required for tissue characterization, but not CE-MRA. In this child it was possible to perform the conventional BH-MRA sequence during free breathing and compare with FB-TRA. The local research ethics committee approved the study and written consent was obtained from all subjects/guardians.

### Imaging protocol

Imaging was performed on a 1.5 Tesla MR scanner (Avanto, Siemens Medical Solutions, Erlangen, Germany) using two spine coils and one body-matrix coil (giving a total of 12 coil elements). A 20–22 gauge plastic intravenous cannula was placed in the subject’s antecubital vein for administration of contrast agent. BH-MRA was performed as part of the clinical scan and FB-TRA was performed at the end of the clinical scan. The interval between the two scans was 29 ± 10 min (range: 10 to 48 min). The same contrast injection protocol was used for each scan; 0.2 mL/kg of Gadoteric acid (Dotarem, Guerbet, Roissy, France) up to a maximum of 10 mL, being injected at a rate of 2 mL/s. The specifics of the two MRA sequences are detailed below.

### Breath-hold MRA sequence

BH-MRA was performed using a 3D Cartesian spoiled gradient echo (SPGR) sequence, acquired in the sagittal orientation. This sequence was accelerated with GRAPPA in the phase encode direction (full parameter details in Table 1). Optimal timing was ensured through the use of a 2D thick slab SPGR bolus tracking sequence. This sequence allowed visualization of contrast as it passed through the heart and great vessels, allowing the BH-MRA to be triggered when the contrast entered the relevant anatomy. In most patients, both left and right heart visualization was required and two angiograms (~13.5 s breath-hold each) were acquired after a single injection of contrast agent, with a 15 s pause between them. In a minority of patients who clinically required visualization of just one vascular bed, only pulmonary or aortic angiograms were acquired.

**Table 1:** Sequence parameters for the BH-MRA and FB-TRA sequences

	BH-MRA	FB-TRA
TE/TR (ms)	~0.8/1.9	~1.5/9.1
Readouts	Cartesian	Spiral
Spiral interleaves for fully sampled *kx-ky*	-	16 Spiral
Cardiac gating	ECG	None
Acceleration factor (in *kx-ky*)	2 (GRAPPA)	4 (SENSE)
Partial-Fourier in *ky*	75 %	-
Matrix size	~144×256	196×196
Image FOV (mm)	~250×435×260	450×450×220
Orientation	Sagittal	Transverse
Number of slices	~144	96
Slice thickness (mm)	~2.0	2.3
Flip angle	25^o^	25^o^
Pixel bandwidth (Hz/pixel)	1500	2170
Acceleration factor (in *kz*)	-	2 (SENSE)
Partial-Fourier in *kz*	75 %	75 %
Breath-hold duration (s)	~13.5	Free-breathing
Measurements	2 per vasculature of interest(1 pre-contrast, 1 post-contrast)	50
Spatial resolution (mm)	~1.7×1.7×1.8	~2.3×2.3×2.3
Temporal resolution (s)	~13.5	~1.3
Total acquisition time (m:s)	~2:30	~1:25

### Free-breathing TRA sequence

FB-TRA was performed using an in-house 3D stack-of-spirals SPGR sequence acquired in the transverse orientation. A uniform density spiral k-space filling strategy was used in *kx-ky* (readout duration ~5 ms), with 16 interleaves required to fill k*-*space at each of the 96 *kz* positions. In order to accelerate the acquisition, *kx-ky* data was undersampled by a factor of four and *kz* was undersampled by a factor of two. In addition, partial Fourier (75 %) was applied along *kz.* The sampling pattern in *kx-ky* was rotated by one position for each acquired *kz* position, in order to reduce artifacts [14]. This undersampled data was reconstructed online using an iterative non-Cartesian 3D SENSE algorithm [15], combined with a homodyne reconstruction [16]. In order to calculate the coil sensitivities from the data itself, the sampling pattern had to be rotated by one position in *kx-ky* for each volume and shifted by one position in *kz* every fourth volume. Combining eight consecutive volumes resulted in a fully sampled central 50 % of k-space, from which the coil sensitivities were calculated by dividing the corresponding image data by the sum of squares of all the coil data [17]. The necessary ‘reference data’ for the homodyne reconstruction was taken from the central *kz* positions of the acquired data [16]. The total acceleration factor achieved was 10.7x and enabled acquisition of an acceptable spatial resolution volume (~2.3×2.3×2.3 mm) every ~1.3 s. All sequence parameters can be seen in Table 1.

### Image analysis

All image data was analyzed using the OsiriX open source DICOM viewing platform (Osirix 5.9, OsiriX foundation, Switzerland) [18]. The BH-MRA and FB-TRA data for each patient were separately anonymized using a random number identifier. All observers were blinded to the patient identity, the other MR data acquired as part of the clinical scan and the results of other diagnostic examinations. The specific image analysis procedures are described below.

### Image quality

Quantitative image quality was assessed by measuring coefficient of variance (CoV), signal-to-noise ratio (SNR), contrast-to-noise ratio (CNR), relative contrast (RC) and edge sharpness (by J.A.S, 7 years experience). The CoV and RC required measurement of the mean and standard deviation of signal intensities (SI, σ) in the blood pool and in a non-enhancing tissue. The blood pool measures were made using vessel regions-of-interest (ROI’s) placed at the sinotubular junction (Ao1) and main pulmonary artery (MPA) from the frame with the highest contrast. The spinal fluid (which is a non-contrast enhancing tissue) was used to make tissue measures. Vessel and tissue CoV and RC were then calculated as follows:

$$ \begin{array}{l}Co{v}_{vessel}=\frac{\sigma_{vessel}}{S{I}_{vessel}}\\ {}Co{v}_{tissue}=\frac{\sigma_{tissue}}{S{I}_{tissue}}\\ {}RC=\frac{\left(S{I}_{vessel}-S{I}_{tissue}\right)}{\left(S{I}_{vessel}+S{I}_{tissue}\right)}\end{array} $$

True quantification of SNR and CNR in images acquired using non-Cartesian parallel imaging is non-trivial, due to the uneven distribution of noise [19]. However, noise can be was estimated as σ_tissue_ allowing SNR and CNR to be calculated using the formula below [20,21];

$$ \begin{array}{l}SNR=\frac{S{I}_{vessel}}{\sigma_{tissue}}\\ {}CNR=\frac{\left(S{I}_{vessel}-S{I}_{tissue}\right)}{\sigma_{tissue}}\end{array} $$

Quantitative edge sharpness (ES) was calculated (from the frame with visually the highest contrast) by measuring the maximum gradient of the normalized pixel intensities across the border of the vessel of interest as previously described [22]. ES was calculated from multiplanar reformatted cross sectional images at six equidistant positions along the thoracic Aorta, and six positions along the pulmonary vasculature (four equidistant positions along the MPA and one in each of the branch PA’s).

#### Diagnostic accuracy

Assessment of the diagnostic accuracy of the two angiographic sequences was performed by two evaluators (V.M. with 12 years CMR experience, and B.P. with 4 years CMR experience) who were not involved in the clinical reporting of the CMR scans for the subjects in this study. Data from each angiographic sequence was separately consensus reviewed in a randomized order using multiplanar reformatting. It should be noted that all frames of the FB-TRA reformats were assessed. Six arterial segments (Aortic root (AoR), aortic arch (AoA), Descending aorta (DescAo), main pulmonary artery (MPA), right pulmonary artery (RPA) and left pulmonary artery (LPA)) were specifically assessed for the presence of stenosis and dilation. In addition, any other positive diagnoses were noted. The diagnosis from the angiographic data was compared to the diagnosis as stated in the clinical CMR report (as assessed from the whole CMR examination, including 3D whole heart imaging and selected cine, black blood and flow imaging).

#### Vessel measurements

One observer (V.M.) measured aortic and pulmonary artery diameters from multiplanar reformats, derived from the BH-MRA and FB-TRA. The FB-TRA multiplanar reformats had an additional temporal dimension and diameters were measured in the frame in which the vessel of interest had the greatest contrast and were displayed most sharply. Aortic diameter was assessed in three positions; the sinotubular junction (Ao1), the proximal descending aorta (Ao2) and descending aorta at the level of the diaphragm (Ao3). Pulmonary artery diameters were assessed in the MPA, the mid LPA and the mid RPA. Where a stenosis was present, the vessel diameter measurements were made at the position of the narrowing. Intraobserver variability (by V.M., > 7 days between measurements) of vessel diameter measurements from both sequences was assessed in a subset of 10 patients, who had both aortic and pulmonary BH-MRA. Additionally, interobserver variability of vessel diameter measurements was performed in these 10 patients (by V.M. and a second observer, B.P.).

### Statistical analysis

All statistical analysis was performed using GraphPad Prism (GraphPad Software Inc., San Diego, CA). The results are expressed as the mean ± standard-deviation. Paired t-tests were used to compare BH-MRA and FB-TRA, in terms image quality and vessel diameter measurements. Additionally, correlation coefficients were calculated.

Diagnostic accuracy was assessed by calculation of the sensitivity and specificity of the BH-MRA and FB-TRA sequences, for the detection of stenosis and dilation. A Fisher’s exact test was used to assess if there were any significant differences in the diagnostic accuracy of the two techniques. The McNemar chi-squared statistical test was used to assess if there were any significance differences in the sensitivity or specificity of the two techniques.

Bland-Altman analysis was performed to give measures of agreement between the vessel diameter measurements from the two sequences, as well as inter and intra observer agreement [23]. One-way ANOVA tests were used to compare the difference in vessel diameter measurements from the two techniques, between all vessel segments. A P-value of less than .05 indicated a significant difference.

## Results

The median age of the patients enrolled in the comparative arm of this study was 23.1 ± 15.7 years (range: 8 to 80 years, 13 of whom were less than 18 years old). The cardiovascular diagnoses in these patients were; repaired tetralogy of Fallot (*n* = 7); hypertrophic cardiomyopathy (*n* = 6); Marfan syndrome (*n* = 6); transposition of the great arteries, post arterial switch (*n* = 3), post atrial switch (*n* = 3); repaired coarctation of the aorta (*n* = 5); pulmonary stenosis (*n* = 3); repaired ventricular septal defect (*n* = 3); dilated aortic root (*n* = 2); repaired anomalous pulmonary venous drainage (*n* = 1); repaired atrial septal defect (*n* = 1); repaired truncus arteriosus (*n* = 1); cor triatriatum (*n* = 1); Ebstein’s anomaly (*n* = 1); subaortic stenosis (*n* = 1); and dilated right ventricle (*n* = 1).

FB-TRA data was successfully acquired in all 45 patients. In 33 patients both pulmonary and aortic BH-MRA’s were acquired, in 10 patients only an aortic BH-MRA was clinically indicated, and in the remaining two patients only a pulmonary BH-MRA was indicated. All subjects were able to follow breath-holding instructions.

The child in whom a free breathing BH-MRA (in both the pulmonary and aortic vasculature) was acquired was 10 years old and had a diagnosis of dilated cardiomyopathy.

### Image quality

**Fig. 1:**
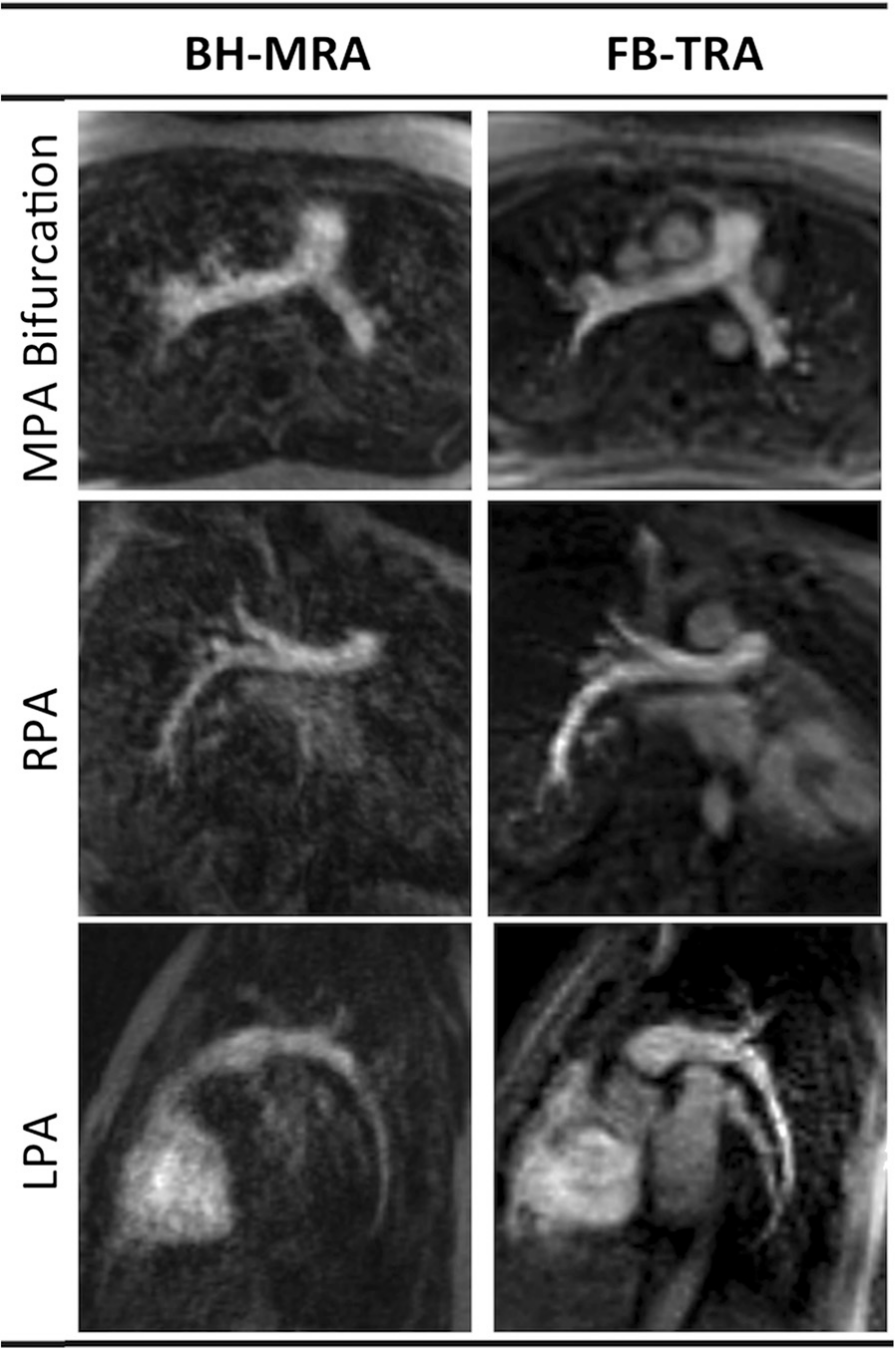
Example multiplanar reformatted image quality from BH-MRA acquired during free-breathing, and FB-MRA in a 10 year old patient with cardiomyopathy

Figure 1 shows images acquired using the BH-MRA sequence but during free breathing. It should be noted that there is a loss of vessel edge sharpness and increased artifact due to respiratory motion. This is compared to the FB-TRA in the same patient, which has better edge definition and very little respiratory artifact (Fig. 1).

**Fig. 2:**
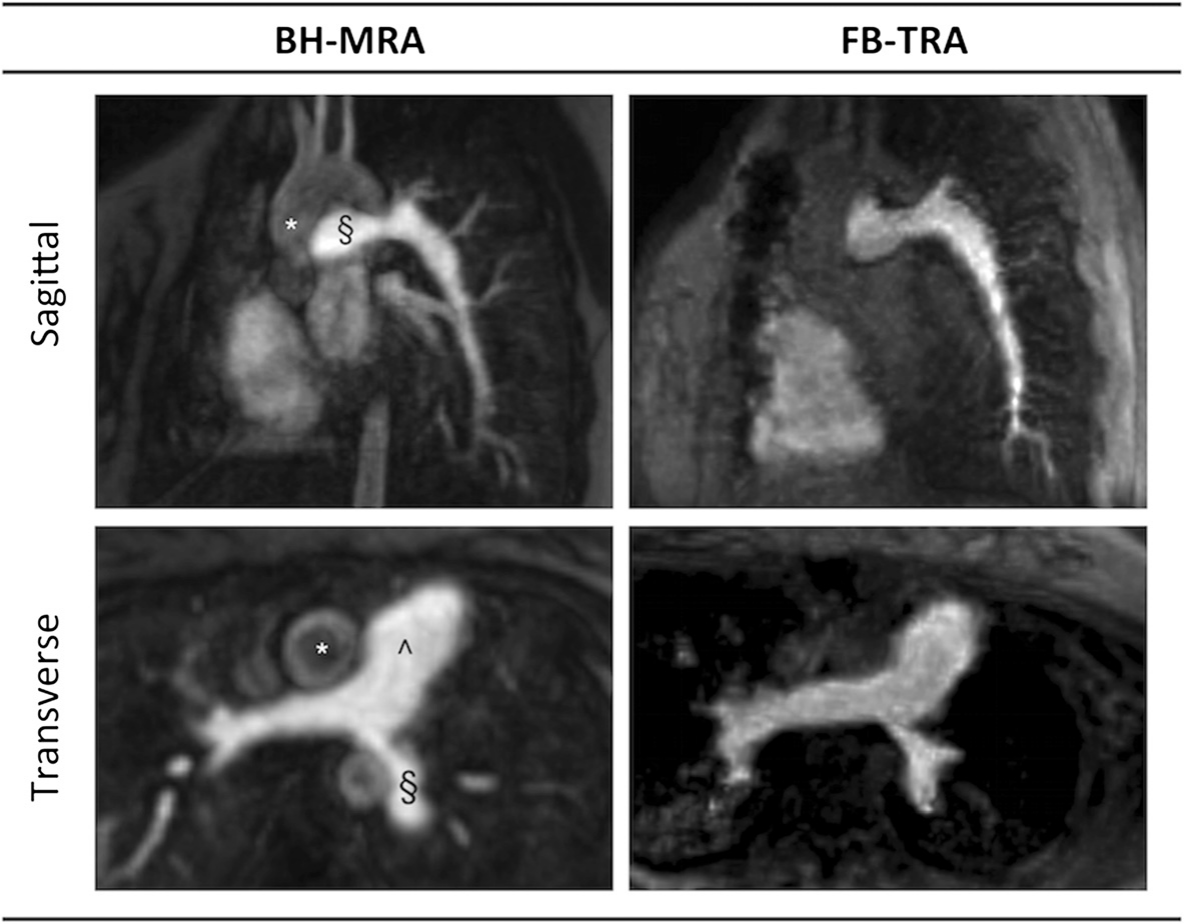
Example multiplanar reformatted image quality from one 16 year old patient with an LPA stenosis, from both BH-MRA and FB-TRA. * Ascending Aorta, § Left Pulmonary Artery, ^ Main Pulmonary Artery

**Fig. 3:**
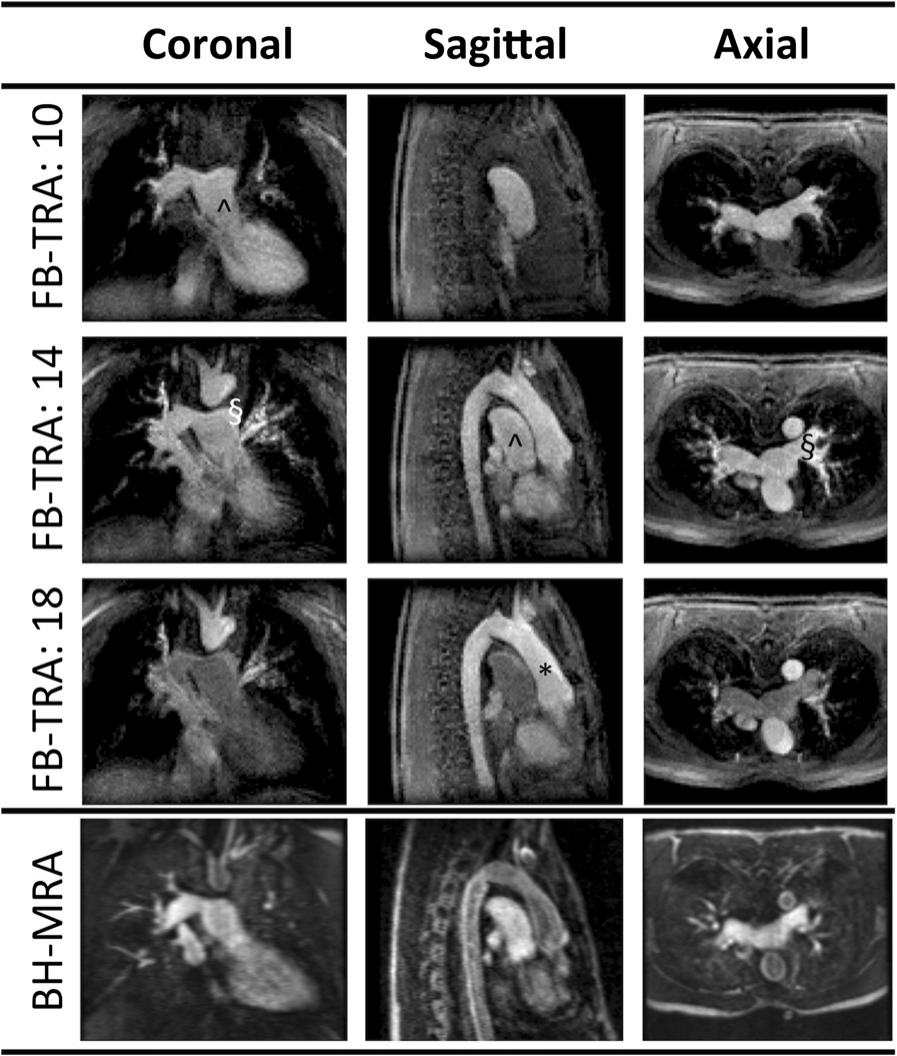
Example image quality in multiple frames (denoted by the numbers in the table header) from the FB-TRA sequence compared to the BH-MRA sequence, in one 29 year old patient whom has undergone an atrial switch. The full movie can be seen online in Additional file 1. * Ascending Aorta, § Left Pulmonary Artery, ^ Main Pulmonary Artery

**Fig. 4:**
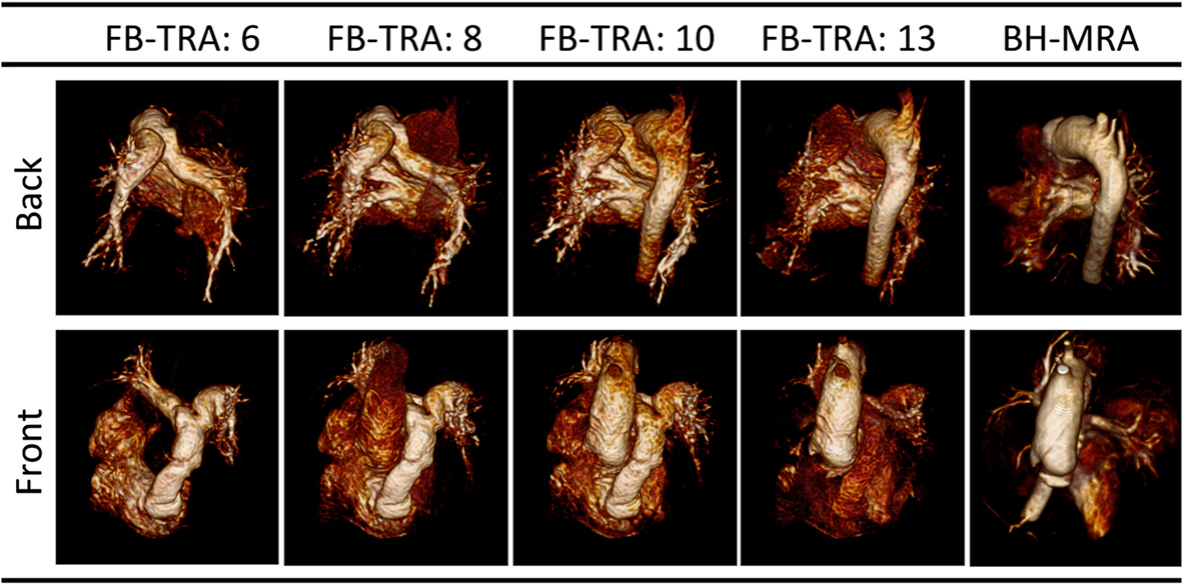
Multiple frames from a 3D reconstruction of the FB-TRA images (denoted by the numbers in the table header) compared to the BH-MRA sequence, in one patient showing kinking of the LPA. The full movie can be seen online in Additional file 2

In the comparative arm of the study, the image quality of both MRA sequences was good. The FB-TRA images contained some residual aliasing resulting from the high acceleration factor used, however these artifacts were mostly in the outer portions of the images. Figure 2 shows examples of the multiplanar reformatted image quality from the two sequences, in one 16 year old patient with an LPA stenosis. Figure 3 shows multiple frames from the FB-TRA sequence, compared to the BH-MRA sequence, in one 29 year old patient, who has an atrial switch – the full FB-TRA movie can be seen online in Additional file 1. Figure 4 shows multiple frames from a 3D reconstruction of the FB-TRA images, compared to the BH-MRA sequence, in one 41 year old patient showing kinking of the LPA – the full FB-TRA movie can be seen online in Additional file 2.

Quantitative image quality results are shown in Table 2. Signal homogeneity (as measured using CoV) in the non-enhancing tissue was found to be significantly higher in the BH-MRA images compared to the FB-TRA images (*P* < 0.0001), however the vessels were found to have a similar homogeneity in both techniques (*P* = 0.52). The BH-MRA images had significantly higher SNR (*P* < 0.0001), CNR (*P* < 0.0001) and RC (*P* = 0.02) compared to the FB-TRA images. However, average edge sharpness was significantly (*P* < 0.0001) higher in the FB-TRA images compared to the BH-MRA images, although the standard deviation of ES was significantly higher in the FB-TRA images (*P* < 0.0001).

**Table 2:** Quantitative image quality results for the BH-MRA and FB-TRA sequences

	BH-MRA	FB-TRA	*P*-value
Signal Intensity: Vessel	156.5 ± 66.4	67.7 ± 14.5*	*P* < 0.0001
Standard Deviation: Vessel	36.9 ± 21.0	15.4 ± 5.2*	*P* < 0.0001
Coefficient of Variation: Vessel (%)	23.1 ± 5.7	22.6 ± 5.0	*P* = 0.52
Signal Intensity: Tissue	20.5 ± 7.9	11.7 ± 3.4*	*P* < 0.0001
Standard Deviation: Tissue	7.2 ± 2.6	7.1 ± 2.2	*P* = 0.85
Coefficient of Variation: Tissue (%)	36.0 ± 6.6	62.7 ± 19.5*	*P* < 0.0001
SNR	24.0 ± 11.6	10.5 ± 4.2*	*P* < 0.0001
CNR	21.1 ± 11.7	8.8 ± 3.8*	*P* < 0.0001
Relative Contrast	0.74 ± 0.13	0.70 ± 0.08*	*P* = 0.02
Edge Sharpness (mm^−1^)			
Average	0.88 ± 0.39	2.33 ± 1.03*	*P* < 0.0001
Standard deviation	0.14 ± 0.08	0.25 ± 0.19*	*P* < 0.0001

*Value is statistically significantly different from BH-MRA

#### Diagnostic accuracy

Anatomical lesions detected by assessment of the whole CMR as stated in the CMR report are listed in Table 3. The BH-MRA sequence provided the correct overall diagnosis in 37/45 patients (diagnostic accuracy = 82 %), and the FB-TRA sequence in 39/45 patients (diagnostic accuracy = 87 %), with no statistical difference between the two sequences (*P* = 0.77). The diagnostic failures are listed in Table 4. It should be noted that in four patients, both reviewers found the same, incorrect diagnosis in the BH-MRA and FB-TRA sequences. In two cases, dynamic kinking/obstruction were poorly appreciated on both ungated MRA sequences. In the third case, isolated valvar stenosis of a pulmonary homograft was not visible on either angiogram. In the remaining case a baffle leak post atrial switch was missed by the BH-MRA and FB-TRA sequences.

**Table 3:** Patient diagnosis

	No. of patients
Dilated AoR	12
Dilated MPA	10
Dilated DescAo	9
Dilated RPA	9
Dilated LPA	7
Dilated AoA	6
LPA stenosis	6
Atrial switch	3
Arterial switch (Lecompte)	3
DescAo stenosis	2
RPA stenosis	2
AoA stenosis	2
MPA stenosis	1
Ebsteins	1
Cor Triatrium	1
Muscular VSD	1
SV defect corrected with LA baffle	1
PDA	1
Absent LSCA	1
Aberrant RCA	1

**Table 4:** Misdiagnosis

BH-MRA	FB-TRA	Actual diagnosis
Proximal DescAo stenosis	Normal	Proximal DescAo stenosis
Normal	Stenosis in arch	Stenosis in arch
Cor Triatrium	None	Cor Triatrium
Normal	SVC baffle stenosis	SVC baffle stenosis
Normal	Mild Proximal LPA stenosis	Mild Proximal LPA stenosis
Normal	Dilated MPA	Dilated MPA
None	None	SVC baffle leak
Normal	Normal	Homograft stenosis
Normal	Normal	LPA stenosis
Normal	Normal	RPA stenosis

The specific ability of the two sequences to assess stenosis (*N* = 13) or dilation (*N* = 53) in the imaged vessel segments (*N* = 234) was also assessed. Overall, the BH-MRA sequence provided the correct diagnosis in 228/234 segments (diagnostic accuracy = 97 %) and the FB-TRA in 230/234 segments (diagnostic accuracy = 98 %), with no statistical difference between the groups (*P* = 0.75). The sensitivity of BH-MRA for specifically identifying stenosis was 62 % compared to 69 % for FB-TRA, with both having a specificity of 100 %. The sensitivity of BH-MRA for identifying dilation was 98 % and of FB-TRA was 100 %, with specificities of 100 % for both sequences. There were no statistical differences between the sequences in terms of sensitivity and specificity to stenosis (*P* = 0.56, *P* = 1.0 respectively) or dilation (*P* = 0.32, *P* = 1.0 respectively).

### Vessel measurements

**Fig. 5:**
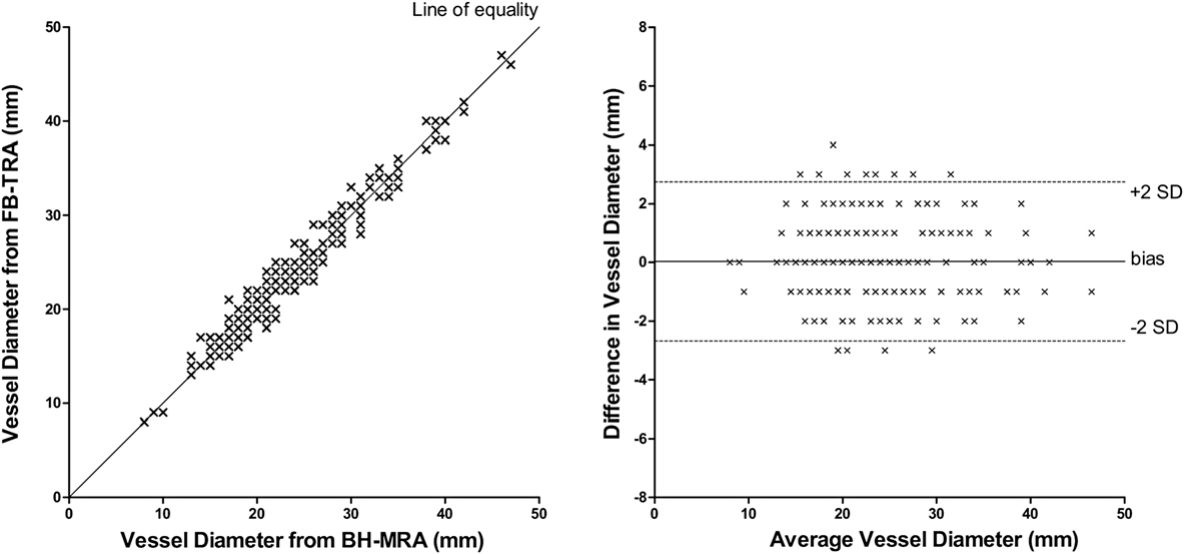
Vessel diameters as measured in all 234 vessels from the BH-MRA and 637 FB-TRA sequences. **a**) Correlation, **b**) Bland-Altman Plot

The vessel diameters were successfully measured in all 234 imaged vessel segments (Ao1, Ao2 and Ao3 in 43 patients, and MPA, RPA and LPA in 35 patients). Scatter and Bland Altman plots for all 234 segments are shown in Fig. 5 with excellent agreement (*r* = 0.98, *P* < 0.05), no bias (0.0 mm, *P* = 0.71), and clinically acceptable limits of agreement (−2.7 to +2.8 mm). There was similarly good agreement between BH-MRA and FB-TRA for each of the individual segments (see Table 5), with one-way ANOVA finding no statistical difference between segments (P = 0.1013).

**Table 5:** Vessel measurements

Vessel	N	BH-MRA (mm)	FB-TRA (mm)	Bias* (mm)	Limits of agreement* (mm)	Correlation coefficient* (r)	*P*-value*
Ao1	43	29 ± 6 (range: 21 to 46)	29 ± 6 (range: 19 to 47)	-0.2	-2.7 to 2.3	0.98	*P* = 0.40
Ao2	43	21 ± 5 (range: 14 to 38)	21 ± 6 (range: 14 to 40)	0.4	-3.0 to 3.7	0.96	*P* = 0.18
Ao3	*43*	19 ± 4 (range: 13 to 32)	19 ± 4 (range: 14 to 34)	0.4	-2.0 to 2.7	0.96	*P* = 0.06
MPA	35	29 ± 7 (range: 17 to 47)	29 ± 7 (range: 16 to 46)	0.1	-2.9 to 3.0	0.98	*P* = 0.82
RPA	35	22 ± 6 (range: 13 to 35)	21 ± 5 (range: 13 to 34)	-0.3	-3.1 to 2.5	0.97	*P* = 0.24
LPA	35	20 ± 5 (range: 8 to 31)	20 ± 5 (range: 8 to 31)	-0.3	-2.5 to 2.0	0.98	*P* = 0.14
Total	234	23 ± 7 (range: 8 to 47)	23 ± 7 (range: 8 to 47)	0	-2.7 to 2.8	0.98	*P* = 0.71

*Calculated with BH-MRA sequence

Inter and intra observer reproducibility showed good agreement of vessel diameters (*r* > 0.988, *P* < 0.0001), with negligible biases (between −0.2 and +0.1 mm) and small limits of agreement (between −2.4 and +2.5 mm). See Table 6 for results.

**Table 6:** Intra and inter observer variability

	Bias (mm)	Limits of agreement (mm)	Correlation (r)
Intraobserver variability			
BH-MRA	−0.2	−2.1 to 1.7	0.99
FB-TRA	0.1	−1.7 to 1.9	0.99
Interobserver variability			
BH-MRA	0.1	−2.4 to 2.5	0.99
FB-TRA	−0.2	−2.4 to 2.1	0.99

## Discussion

We have shown that it is possible to perform free-breathing high spatio-temporal resolution time resolved angiography, in a population of 45 patients with paediatric and adult congenital heart disease. This was achieved through the use of a highly accelerated 3D stack of spirals acquisition with no data sharing between frames. The main findings were; i) FB-TRA was feasible in all patients, ii) FB-TRA had lower SNR, CNR and RC than BH-MRA, iii) FB-TRA had a similar diagnostic accuracy to BH-MRA, and iv) Vessel measurements made with the two sequences were comparable.

### Time resolved angiography

The main aim of this study was to demonstrate that rapid acquisition allows angiographic data to be acquired during free-breathing. Rapid acquisition is the hallmark of time resolved angiography and we employed this approach in the current study. However, unlike the majority of TRA sequences, our implementation did not rely on data sharing techniques such as sliding window reconstruction [12], CENTRA keyhole [11] or TREAT [13]. Instead, we used a combination of efficient k-space filling with spiral trajectories, sensitivity encoding and partial Fourier to achieve rapid acquisition (~1.3 s/volume). The benefit of this approach is that data used for reconstruction of each volume only contains a limited amount of respiratory motion. We were able to show that this resulted in better image quality compared to conventional MR angiography performed during free breathing (duration ~13 s). Slightly unexpectedly, we also found that FB-TRA images had significantly higher average edge sharpness than BH-MRA images. This is probably due to BH-MRA including more cardiac motion (10–15 heart beats) than FB-TRA (1–2 heart beats) resulting in more edge blurring, despite the breath-hold.

Highly undersampled spiral imaging does have some disadvantages. The main drawback is increased signal inhomogeneity due to spiral off-resonance effects, trajectories errors and data undersampling [27]. In our study, FB-TRA images had significantly lower SNR, CNR and tissue signal homogeneity compared to BH-MRA images. However, vessel signal homogeneity was similar for both sequences. This was probably due to less variation in contrast concentration during the rapid FB-TRA acquisition (~1.3 s) compared to the longer BH-MRA acquisition (~13.5 s), which would compensate for the increased noise. In addition, RC was only marginally higher in BH-MRA, demonstrating that there are only small differences in the tissue contrast provided by these two sequences. As will be discussed later, the similarity of these metrics may explain the comparable diagnostic accuracy. Alternative reconstruction algorithms could also be investigated to improve the image quality of the FB-TRA sequence. The most promising is compressed sensing (CS), which is well suited to angiography due to the inherent sparseness of the data [24]. However, CS reconstructions are more computationally intensive than our proposed approach and could only be justified if they significantly improved image quality.

Another problem with spiral imaging relates to the orientation of the imaging slab. In Cartesian imaging, readout oversampling reduces artifact from signal outside the FOV in the frequency encode direction. This allows the BH-MRA to be acquired in the sagittal orientation for optimization of coverage, acquisition time and coil placement for parallel imaging. In spiral imaging there is no single ‘readout direction’, preventing oversampling being used to reduce artifact. Thus, FB-TRA data had to be acquired in the transverse orientation to reduce the amount of signal outside the FOV. Although this may not be the optimal orientation in terms of coverage, this limitation is offset by the greater efficiency and possible undersampling available with spiral imaging.

### Diagnostic accuracy

The FB-TRA and BH-MRA were found to have statistically comparable overall diagnostic accuracy, as well as similar sensitivity and specificity for detection of stenosis and dilatation. This demonstrates that although SNR and CNR were lower in FB-TRA images, diagnostic accuracy was not significantly affected. However, it should be noted that both MRA sequences missed lesions in four out of 45 patients. In all these cases, MRA failed because the lesions were dynamic, membranous or intra-cardiac, situations where ungated imaging is known to struggle [25]. This justifies the use of multiple CMR sequences when attempting to make a comprehensive diagnosis in CHD.

In terms of vessel measurements, there was also excellent agreement between FB-TRA and BH-MRA, with no bias and clinically acceptable limits of agreement. Furthermore, there was no difference in intra and inter observer variability. This suggests that despite the reduction in image quality, FB-TRA allows accurate and reproducible quantitative and qualitative assessment of vascular structure. This may partly be related to the fact that RC and CoV_vessel_, which both specifically relate to vessel visualization, were similar in the FB-TRA and BH-MRA sequences. In addition, improved edge sharpness may compensate for any reduction in SNR and CNR.

### Clinical utility

The main clinical advantage of our technique, is that it can be performed during free-breathing. This opens up the possibility of performing MRA in patients who are unable to breath hold. However, in this study we chose patients who were able to comply with breath-hold instructions for two reasons. Firstly, at our institution children who require vascular assessment, but cannot breath hold, undergo CMR under general anaesthetic. This makes it difficult to perform a comparative study of BH-MRA and FB-TRA in this population. Secondly, to demonstrate the comparable utility of FB-TRA, it was essential that the BH-MRA be performed in an optimal way (*i.e.* during a breath hold). Thus, further studies are required to investigate the utility of FB-TRA in small children (<8 years). In particular, the requirement for higher resolution imaging (with the commensurate loss of SNR and CNR) and problems with higher respiratory rates would have to be addressed.

A further benefit of time resolved MRA sequences, is that it is not necessary to calculate exact bolus timing [10]. This may significantly simplify workflow, helping to reduce overall scan times and increase throughput. Finally, time resolved MRA also allows assessment of perfusion kinetics [26], which although not specifically assessed in this study, may be beneficial in certain groups of patients.

### Limitations

In this study, the BH-MRA sequence was always performed before FB-TRA sequence (~29 min between the two MRA scans). This was done to ensure that the clinically indicated BH-MRA was not affected by residual contrast (mean half-life of Dotarem is ~2.0/1.4 h in male/female subjects). However, this means that the FB-TRA may have been adversely affected by the contrast given for the BH-MRA. Thus, the results presented here give a conservative estimation of the FB-TRA technique in terms of image quality and diagnostic accuracy.

It was not possible to truly anonymize the BH-MRA data from the FB-TRA data, due to the temporal dimension of the TRA data. This meant that it was not possible to compare subjective image quality measures from the two sequences, as it is likely to be influenced by observer bias.

A final limitation of this technique is that the current online reconstruction is time consuming, taking approximately one hour for all 50 frames of FB-TRA data. However, the development of new highly parallel architectures, such as graphical processing units, should be able to significantly speed this up, as the pixel-wise calculations can be parallelized [28]. Alternatively, the reconstruction time could be reduced with the use of a bolus tracking sequence to visualize the contrast arriving in vasculature of interest, and trigger the start of the FB-TRA sequence, thereby reducing the amount of data acquired.

## Conclusions

To conclude, we have described a free-breathing, time-resolved 3D spiral MRA technique that has been shown to enable accurate diagnosis and vessel measures compared to conventional breath-hold, Cartesian MRA. This technique simplifies the MRA technique and will enable angiography to be performed on children and adults in whom breath-holding is difficult.

## Additional files

Additional file 1:
**Movie showing FB-TRA sequence in one 29 year old patient whom has undergone an atrial switch.** Left: Coronal, Middle: Sagittal, Right: Axial. Additional file 2:
**Movie showing 3D reconstruction of FB-TRA images, in one 41 year old patient, showing kinking of the LPA.** Left: Front view, Right: Back view.

## Abbreviations

2D: Two-dimensional
3D: Three-dimensional
Ao1: Sinotubular junction
Ao2: Proximal descending aorta
Ao3: Descending aorta at the level of the diaphragm
AoA: Aortic arch
AoR: Aortic root
BH-MRA: Breath-hold magnetic resonance angiography
CE-MRA: Contrast enhanced magnetic resonance angiography
CENTRA: Contrast-enhanced timing-robust angiography
CHD: Congenital heart disease
CMR: Cardiovascular magnetic resonance
CNR: Contrast-to-noise ratio
CS: Compressed Sensing
DescAo: Descending aorta
FB-TRA: Free-breathing time resolved magnetic resonance angiography
FOV: Field-of-view
LA: Left atrium
LPA: Left pulmonary artery
LSCA: Left subclavian artery
MPA: Main pulmonary artery
PDA: Patent ductus arteriosus
RC: Relative contrast
RCA: Right coronary artery
ROI: Region-of-interest
RPA: Right pulmonary artery
SENSE: Sensitivity encoding
SI: Signal intensity
SNR: Signal-to-noise ratio
SV: Single ventricle
TRA: Time resolved magnetic resonance angiography
TREAT: Time-resolved echo-shared angiographic technique
VSD: Ventricular septal defect
